# Muscle biopsy long-chain omega-3 polyunsaturated fatty acid compositions, IMF and FMP in Australian pasture-based Bowen Genetics Forest Pastoral Angus, Hereford, and Wagyu Beef Cattle

**DOI:** 10.1186/s12917-024-03906-2

**Published:** 2024-03-09

**Authors:** John Roger Otto, Felista Waithira Mwangi, Shedrach Benjamin Pewan, Oyelola Abdulwasiu Adegboye, Aduli Enoch Othniel Malau-Aduli

**Affiliations:** 1 School of Environmental and Life Sciences, College of Engineering, Science and Environment, The University of Newcastle, Callaghan, NSW 2308 Australia; 2 School of Medicine and Public Health, College of Health, Medicine and Wellbeing, The University of Newcastle, Callaghan, NSW 2308 Australia; 3 National Veterinary Research Institute, Private Mail Bag 01 Vom, Jos, Plateau State Nigeria; 4 Menzies School of Health Research, Charles Darwin University, Casuarina, NT 0811 Australia

**Keywords:** Health-beneficial fatty acids, Intramuscular fat, Fat melting point, Meat eating quality, *M. longissimus dorsi*, Gender, Bowen Genetics Forest Pastoral Wagyu, Angus, Hereford

## Abstract

**Background:**

We investigated breed and gender variations in the compositions of long-chain (≥ C20) omega-3 polyunsaturated fatty acids (LC omega-3 PUFA), fat melting point (FMP) and intramuscular fat (IMF) contents in biopsy samples of the *M. longissimus dorsi* muscle of grazing beef cattle. The hypothesis that biopsy compositions of health-beneficial LC omega-3 PUFA, FMP and IMF in a pasture-based production system will vary with breed, was tested. Muscle biopsies were taken from 127 yearling pasture-based Angus, Hereford, and Wagyu heifers and young bulls exclusive to the Australian Bowen Genetics Forest Pastoral breeding stud averaging 12 ± 2.43 months of age and under the same management routine.

**Results:**

Breed had a significant influence on IMF, FMP, and the compositions of oleic acid, α-linolenic acid (ALA), eicosapentaenoic (EPA), docosahexaenoic (DHA), docosapentaenoic (DPA), and total EPA + DHA + DPA in the *M. longissimus dorsi* muscle biopsies (*P* ≤ 0.03). The Wagyu breed had the highest (11.1%) and Hereford the lowest (5.9%) IMF (*P* = 0.03). The reverse trend was observed in FMP values where the Hereford breed had the highest (55 °C), Angus intermediate (46.5 °C), and Wagyu the lowest (33 °C) FMP. The Wagyu and Angus breeds had similar oleic fatty acid (18:1n-9) content, while the Hereford breed had the lowest (*P* < 0.01). The highest ALA, DPA, total EPA + DHA, total EPA + DHA + DPA and total ALA + EPA + DHA + DPA contents were detected in the Wagyu breed (*P* ≤ 0.03). The Hereford had similar EPA and DPA contents to the Angus (*P* ≥ 0.46). Total EPA + DHA + DPA contents in Wagyu, Angus, and Hereford were 28.8, 21.5, and 22.1 mg/100g tissue (*P* = 0.01), respectively. Sex was an important source of variation that influenced LC omega-3 PUFA composition, FMP and IMF, where yearling heifers had higher IMF (11.9% vs 5.3%), lower FMP (33°C vs 37°C), and higher LC omega-3 PUFA than bulls.

**Conclusion:**

All the results taken together indicate that the Wagyu breed at 28.8 mg/100g tissue, was the closest to meeting the Australia and New Zealand recommended source level threshold of 30 mg/100g tissue of health-beneficial ≥ C20 omega-3 FA content. Since gender was a significant determinant of LC omega-3 PUFA composition, IMF content and FMP, it should be factored into enhancement strategies of healthy meat eating quality traits in grazing cattle. These findings also suggest that the Bowen Genetics Forest Pastoral beef cattle studs are important sources of LC omega-3 PUFA that can be used to cover the deficit in these health claimable fatty acids in Western diets.

## Background

Consumers perceive consistency in meat eating quality as a critical driver of beef consumption, hence beef producers and processors continuously strive to add value to ruminant-derived meat and meat products to meet this rising market demand [1–5]. Red meat plays a vital role in meeting human dietary requirements, because it provides high quality protein, fat-soluble vitamins, minerals, and essential fatty acids [6, 7]. Red meat is also a source of health-beneficial Long Chain (≥ C20) omega-3 polyunsaturated fatty acids (LC omega-3 PUFA), comprising eicosapentaenoic (EPA, 20:5n-3), docosahexaenoic (DHA, 22:6n-3), and docosapentaenoic (DPA, 22:5n-3) acids. EPA, DHA and DPA are known to positively influence human health with mitigating properties against the prevalence of metabolic, cardiovascular, and chronic diseases [8–10], hence the need for striking the right balance between health-beneficial LC omega-3 PUFA composition and intramuscular fat (IMF) content in bovine meat is essential. The research quest for healthy red meat with enhanced culinary properties of sensory organoleptic attributes of taste, aroma, tenderness, juiciness, and health-beneficial LC omega-3 PUFA is on the increase [11–13]. However, humans cannot synthesize LC omega-3 PUFA due to their inability to produce Δ12- and Δ15-desaturase enzymes [14, 15]. As a result, humans rely heavily on dietary sources like red meat and seafood to meet their daily dosage of LC omega-3 PUFA [9, 15, 16]. Seafood, comprising mainly oily fish such as mackerel, herrings, sardines, salmon, and tuna, remains a key source (80%) of LC-omega-3 PUFA. However, due to limited access to fish and other seafood products, the focus has shifted to increasing and developing a variety of alternative food sources, especially of animal origin, necessitated by the nutritional and health claims for functional foods rich in LC-omega-PUFA. These include processed and raw foods enriched with LC-omega-3 PUFA from meat, milk, and eggs from livestock fed omega-3 oil rich supplements [17–22].

However, over-exploitation of global wild fish stocks threatens the sustainability of seafood as a source of LC omega-3 PUFA [23], while the low availability of seafood in many parts of the world limits consumption [24, 25]. As a result, many consumers do not meet the recommended seafood intake. For instance, Australians consumed 245 g of seafood per week in 2019–2020 [26], which is 135 g below the 375 g recommended by the Food Standards Australia New Zealand [27]. Therefore, red meat will be an important alternative source of LC omega-3 PUFA for Western diets due to preference and culture inculcation [28]. A large contribution of total LC-omega-3 PUFA intake in adult Australians comes from beef and lamb at 28%, compared to poultry and pork at 10% and 4%, respectively [29]. For most Australians, meat is the major contributor of LC-omega-3 PUFA, especially DPA, the predominant PUFA in meat [29–31]. Therefore, recognizing the potential significance of meat consumption as a major contributor to dietary intakes of LC-omega-3 PUFA in the Australian diet, this paper sought to explore and unravel the breed variations in LC omega-3 PUFA compositions, IMF and FMP in grazing purebred Angus, Hereford and Wagyu beef cattle.

The beef industry contributes significantly to the Australian economy. Australia produced 1.9 million tonnes of carcass weight in 2021, and was the fourth largest beef and veal exporter after Brazil, India, and the USA [32]. Beef and veal consumption in Australia was 18.1 kg/capita beef carcass weight equivalent in 2020 compared to the global average of 6.3 kg/capita [33]. To meet the market demand while minimizing environmental impacts, the Australian beef production systems target suitable cattle genotypes and high productivity to maximize income and minimize input costs, mainly feed costs, which may exceed 60% of the total production costs [32, 34]. Therefore, beef cattle production in Australia is mainly pasture-based. Grasslands occupy approximately 60% of the land surface, of which beef cattle constitute 78% of the main grazing livestock population [35, 36]. It has been reported that beef production can be sustainable where pastures are the primary feed source [34]. A sustainable beef production system must have a low environmental impact, contribute to food security, maintain biodiversity in the ecosystem, is easily accessible, and economically affordable [34]. A pasture-based production system fits this bill because it provides energy and essential nutrients at approximately half the cost of grain-based feedlot rations, alongside an efficient utilization of beef genotypes for high productivity, maximum income and limited input costs, thus ensuring the most cost-effective means of achieving maximum and efficient beef production sustainably [34, 37]. Pastures generally contain a higher proportion of α-linolenic acid (ALA, 18:3n-3), which is a precursor to the synthesis of the health-beneficial LC omega-3 PUFA [38, 39], than grain-based rations in the feedlot system.

Current research focus in the red meat industry has shifted towards meat eating quality with emphasis now being placed on increasing LC omega-3 PUFA and IMF on one hand, while lowering FMP and saturated fatty acids (SFA) on the other, because SFA serve as precursors for cholesterol and low-density lipoproteins (LDL) [5, 11, 40–44]. Past investigations have suggested that nutritional strategies can be used to increase beneficial LC omega-3 PUFA and IMF contents, and reduce SFA in beef [45]. However, the bioavailability of supplemented omega-3 in the muscle is significantly affected by biohydrogenation in the rumen [46, 47]. Conversely, previous studies have also demonstrated the genetic enhancement of LC omega-3 PUFA in the *M. longissimus dorsi* muscle of beef cattle through breed selection [48, 49]. Therefore, the genetic selection of beef cattle breeds with greater genetic propensities to synthesize LC omega-3 PUFA offer a more permanent, long-term, and cumulative approach to modifying fatty acid composition in ruminants [50, 51]. Furthermore, there is evidence of regional and production system variations in the fatty acid compositions of Angus, Hereford, Limousin, Longhorn, and Wagyu beef cattle raised in Japan and the USA [40, 42, 43, 52–56]. However, most of these previous beef cattle fatty acid composition studies originated from breeds on diverse diets and of different ages, making it difficult to extrapolate the results and make global comparisons [40, 43].

IMF (also referred to as marbling), is primarily used to evaluate carcass quality in Australia and other developed countries [57–59]. IMF is a major determinant of eating quality and contributes to palatability, tenderness, flavour, juiciness and overall liking in beef [60, 61]. IMF is also directly associated with the accumulation of oleic acid (18:1n-9) [62, 63], while beef flavour is positively influenced by the concentration of oleic acid [12, 64, 65]. FMP is an index of fat hardness or softness in beef [61, 66]. Soft fat has a relatively lower melting point than hard fat, and this quality index has implications on beef processing and eating quality [66, 67]. High oleic acid content in beef IMF decreases FMP, leading to softer fat because of the presence of a double bond in its carbon atom nomenclature [67, 68].

In Australia, Hereford, Wagyu, and Angus cattle are increasingly being used to produce beef with high eating quality characteristics such as tenderness, juiciness, flavour and marbling [34, 60, 61]. However, more studies are required to shed more light on the IMF, FMP and LC omega-3 PUFA compositions in the *M. longissimus dorsi* muscles of Australian pasture-based Angus, Hereford, and Wagyu under the same routine nutritional regime, similar ages and production system for an unbiased comparative analysis of eating quality attributes when the animals are still young and alive. Therefore, the primary objective of this study was to evaluate and compare the compositions of LC omega-3 PUFA, IMF, and FMP in *M. longissimus dorsi* muscle biopsies of Angus, Hereford, and Wagyu beef cattle in a pasture-based production system. It was hypothesized that the compositions of health-beneficial LC omega-3 PUFA, IMF, and FMP will vary between the muscle biopsies of pasture-fed Angus, Hereford, and Wagyu beef cattle raised under the same production system.

## Results

Results from Kruskal–Wallis tests of breed variations in IMF, FMP, and fatty acid profile of the *M. longissimus dorsi* muscle and test of significance (*P* < 0.05) in Angus, Hereford, and Wagyu cattle are presented in Table 1, and sex differences are presented in Table 2. Breed had a significant influence on IMF, FMP, and the compositions of oleic acid, ALA, EPA, DHA, DPA, and total EPA + DHA + DPA of *M. longissimus dorsi* muscle (*P* ≤ 0.03; Table 1), in which the Wagyu and Angus breeds had the highest and Hereford the lowest IMF (*P* = 0.03). The reverse trend was observed in FMP values where Hereford cattle had the highest, Angus intermediate, and Wagyu the lowest FMP.

**Table 1:** Median (inter-quartile range) of fatty acid profiles (mg/100 g tissue), intramuscular fat (%), and fat-melting point (°C) in the *M. longissimus dorsi* of Bowen Genetics Forest Pastoral Angus, Hereford, and Wagyu beef cattle studs

IMF, FMP and Fatty acids	Breed			
	Angus (*n* = 30)	Hereford (*n* = 22)	Wagyu (*n* = 75)	*P*-value
Intramuscular fat (%)	8.0 (4.1–13.3)^b^	5.9 (5.3–6.0)^a^	11.1 (5.5–12.0)^b^	0.03
Fat melting point (^o^C)	46.5 (35.0–47.8)^b^	55.0 (54.0–55.5)^c^	33.0 (29.5–35.3)^a^	< 0.01
Oleic acid (18:1n-9)	444.4 (216.2–863.0)^b^	177.5 (113.0–294.6)^a^	488.6 (241.7–1035.5)^b^	< 0.01
Linoleic acid (LA; 18:2n-6)	57.5 (49.7–72.3)	58.3 (39.1–76.1)	59.9 (45.7–75.8)	0.70
α-Linolenic acid (ALA; 18:3n-3)	13.1 (10.8–15.9)^a^	12.8 (12.0–14.3)^a^	21.5 (11.4–27.3)^b^	< 0.01
EPA^1^-20:5n-3	8.2 (5.9–10.4)^a^	9.0 (7.5–9.6)^ab^	11.5 (5.1–14.0)^b^	0.03
DHA^2^-22:6n-3	1.1 (0.6–1.3)^a^	1.3 (1.0–1.6)^b^	1.5 (0.7–2.2)^b^	0.02
DPA^3^-22:5n-3	11.8 (8.5–12.9)^a^	12.3 (9.2–13.2)^a^	15.1 (7.1–19.4)^b^	0.01
EPA + DHA	9.4 (6.6–11.6)^a^	10.3 (8.7–11.0)^a^	13.1 (5.81–16.3)^b^	0.02
EPA + DHA + DPA	21.5 (16.1–24.4)^a^	22.1 (17.7–24.6)^a^	28.8 (12.6–36.3)^b^	0.01
ALA + EPA + DHA + DPA	33.6 (26.2–42.2)^a^	36.3 (32.5–37.8)^a^	52.6 (24.6–63.8)^b^	< 0.01
*t*SFA^4^	453.1 (167.3–2634.0)^ab^	233.2 (132.9–4105.0)^a^	467.7 (111.0–7007.0)^b^	0.02
*t*MUFA^5^	511.1 (156.7–2831.0)^b^	203.0 (110.5–3866.0)^a^	567.5 (114.0–8760.0)^b^	< 0.01
*t*PUFA^6^	152.3 (52.6–241.6)	135.0 (86.4–264.5)	181.8 (47.9–718.5)	0.11
n-6 PUFA^7^	116.0 (37.8–157.9)	95.3 (58.9–200.3)	118.4 (40.8–609.0)	0.35
n-3 PUFA^8^	37.6 (11.7–80.5)^a^	39.7 (21.4–79.6)^ab^	56.2 (7.0–130.9)^b^	0.01
n-6/n-3^9^	2.8 (2.0–5.8)^b^	2.5 (1.9–4.2)^ab^	2.1 (1.3–6.0)^a^	< 0.01

^1^Eicosapentaenoic

^2^Docosahexaenoic

^3^Docosapentaenoic acid

^4^Total saturated fatty acids (∑SFA = 14:0, 15:0, 16:0, 17:0, 18:0, 20:0, 21:0, 22:0, 23:0, 24:0)

^5^Total monounsaturated fatty acids (ΣMUFA = 14:1 + 16:1n-13t + 16:1n-9 + 16:1n-7 + 16:1n-7t + 16:1n-5c + 17:1n8 + a17:0 + 18:1n-7t + 18:1n-5 + 18:1n-7 + 18:1n-9 + 18:1a, + 18:1b + 18:1c + 19:1a + 19:1b + 20:1n-11 + 20:1n-9 + 20:1n-7 + 20:1n5 + 22:1n-9 + 22:1n-11 + 24:1n-9)

^6^Total polyunsaturated fatty acids (ΣPUFA = 18:4n-3 + 18:3n-6 + 18:2n-6 + 18:3n-3 + 20:2n-6 + 20:3 + 20:3n-6 + 20:4n-3 + 20:4n-6 + 20:5n-3 + 22:4n-6 + 22:5n-3 + 22:5n-6 + 22:6n-3)

^7^Omega-6 polyunsaturated fatty acids (Σn-6 PUFA = 18:2n-6 + 18:3n-6 + 20:2n-6 + 20:3n-6 + 20:4n-6 + 22:4n-6 + 22:5n-6)

^8^Omega-3 polyunsaturated fatty acids (Σn-3 PUFA = 18:3n-3 + 18:4n-3 + 20:4n-3 + 20:5n-3 + 22:5n-3 + 22:6n-3)

^9^Ratio n-6/n-3

Medians in rows bearing different superscripts (a, b, c) differ significantly (*P* < 0.05)

**Table 2:** Gender differences presented as median (inter-quartile range) of fatty acid profiles (mg/100g tissue), intramuscular fat (%), and fat-melting point (°C) in the *M. longissimus dorsi* muscle of Bowen Genetics Forest Pastoral Angus, Hereford and Wagyu beef cattle studs

Characteristic	Yearling Heifer	Yearling Bull	*P*-value
Intramuscular fat (%)	11.9 (6.0 – 12.8)	5.3 (3.2 – 5.9)	< 0.01
Fat melting point (^o^C)	33 (29 – 47)	37 (35 – 54)	< 0.01
Oleic acid (18:1n-9)	930 (452 – 1,317)	224 (142 – 358)	< 0.01
LA^1^-18:2n-6	21 (13 – 27)	14 (12 – 25)	0.17
ALA^2^-18:3n-3	11.6 (8.7 – 14.7)	9.3 (8.3 – 11.6)	0.03
EPA^3^-20:5n-3	1.54 (1.22 – 2.24)	1.29 (1.03 – 1.64)	0.10
DHA^4^-22:6n-3	16.1 (12.2 – 22.7)	12.8 (11.5 – 14.9)	0.01
DPA^5^-22:5n-3	13.1 (9.9 – 16.8)	10.6 (9.5 – 12.8)	0.03
EPA + DHA	29 (22 – 40)	24 (21 – 27)	0.01
EPA + DHA + DPA	52 (38 – 69)	38 (35 – 53)	0.02
ALA + EPA + DHA + DPA	853 (480 – 1,180)	269 (188 – 396)	< 0.01
*t*SFA^6^	1,013 (522 – 1,447)	276 (178 – 422)	< 0.01
*t*MUFA^7^	199 (156 – 218)	153 (123 – 174)	< 0.01
*t*PUFA^8^	135 (116 – 150)	109 (89 – 127)	< 0.01
n-6 PUFA^9^	57 (43 – 77)	42 (38 – 57)	0.01
n-3 PUFA^10^	2.13 (1.92 – 2.67)	2.20 (1.91 – 2.88)	0.48
n-6/n-3^11^	0.48 (0.38 – 0.52)	0.46 (0.35 – 0.52)	0.47

^1^Linoleic acid

^2^α-Linolenic acid

^3^Eicosapentaenoic

^4^Docosahexaenoic

^5^Docosapentaenoic acid

^6^Total saturated fatty acids

^7^Total monounsaturated fatty acids

^8^Total polyunsaturated fatty acids

^9^Omega-6 polyunsaturated fatty acids

^10^Omega-3 polyunsaturated fatty acids

^11^Ratio n-6/n-3

There were variations in fatty acid composition between the cattle breeds (Table 1). The Wagyu and Angus breeds had similar oleic acid (18:1n-9) contents, closely followed by the Hereford (*P* < 0.01). However, no differences were observed between the breeds in LA (18:2n-6) content. The highest ALA, and DPA were detected in Wagyu (*P* ≤ 0.03). Hereford had similar EPA and DPA concentrations to Angus (*P* ≥ 0.46). Total EPA + DHA + DPA content in Wagyu, Angus, and Hereford was 28.8, 21.5, and 22.1 mg/100g tissue (*P* = 0.01), respectively. At 28.8 mg/100g tissue, the Wagyu was the closest to Australia and New Zealand recommended “source level” threshold of 30 mg/100g tissue of health-beneficial ≥ C20 LC omega-3 PUFA content [69, 70].

The effect of gender on fatty acid profile, IMF (%), and FMP (^o^C) of the *M. longissimus dorsi* muscle of Angus, Hereford and Wagyu cattle are presented in Table 2, where it was demonstrated that sex significantly (*P* ≤ 0.03) affected IMF, FMP, and the compositions of oleic acid, ALA, DHA, DPA, EPA + DHA, EPA + DHA + DPA, ALA + EPA + DHA + DPA, total saturated fatty acids (*t*SFA), monounsaturated fatty acids (*t*MUFA), polyunsaturated fatty acids (*t*PUFA), and omega-6 (n-6) PUFA. It was also evident that yearling heifers produced more IMF (11.9% vs 5.3%) with lower FMP (33°C vs 37°C) than yearling bulls. Furthermore, yearling heifers had higher contents of oleic acid (930 vs 224 mg/100g), ALA (11.6 vs 9.3 mg/100g), DHA (16.1 vs 12.8 mg/100g), DPA (13.1 vs 10.6 mg/100g), EPA + DHA (29 vs 24 mg/100g), EPA + DHA + DPA (52 vs 38 mg/100g), ALA + EPA + DHA + DPA (853 vs 269 mg/100g), *t*SFA (1013 vs 276 mg/100g), *t*MUFA (199 vs 153 mg/100g), *t*PUFA (135 vs 109 mg/100g), and n-6 PUFA (57 vs 42 mg/100g), than yearling bulls. However, omega-3 (n-3) PUFA (2.13 vs 2.20 mg/100g) and n-6/n-3 ratio (0.48 vs 0.46) were similar regardless of gender. The interaction between breed and sex was a significant (*P* ≤ 0.05) source of variation in FMP, ALA, EPA, DHA, DPA and EPA + DHA, as demonstrated in Table 3, suggesting that it should be taken into consideration when selecting for health-beneficial LC omega-3 PUFA, FMP and IMF in the *M. longissimus dorsi* muscle of grazing Angus, Hereford and Wagyu beef cattle.

**Table 3:** The effect of breed, sex and their interactions on fatty acid composition, IMF and FMP of Bowen Genetics Forest Pastoral Angus, Hereford and Wagyu beef cattle studs

Characteristics	Beta	Std. Error	*P*-value
***Intramuscular fat (%)***			
Breed (Hereford vs Angus)	0.50	1.41	0.73
Breed (Wagyu vs Angus)	-2.57	1.26	0.05
Sex (Male vs Female)	-7.03	1.53	< 0.01
Breed*Sex	2.07	1.85	0.27
***Fat melting point (°C)***			
Breed (Hereford vs Angus)	15.43	1.70	< 0.01
Breed (Wagyu vs Angus)	-17.12	1.50	< 0.01
Sex (Male vs Female)	-12.36	1.82	< 0.01
Breed*Sex	17.09	2.30	< 0.01
***Oleic acid (18:1n-9)***			
Breed (Hereford vs Angus)	-70.62	339.81	0.84
Breed (Wagyu vs Angus)	477.35	298.19	0.11
Sex (Male vs Female)	-392.58	368.92	0.29
Breed*Sex	-399.46	441.68	0.37
***LA***^***1***^*** (18:2n-6)***			
Breed (Hereford vs Angus)	-0.75	5.12	0.88
Breed (Wagyu vs Angus)	13.64	4.50	< 0.01
Sex (Male vs Female)	0.41	5.56	0.94
Breed*Sex	-3.94	6.66	0.56
***ALA***^***2***^*** (18:3n-3)***			
Breed (Hereford vs Angus)	0.00	1.37	1.00
Breed (Wagyu vs Angus)	5.10	1.21	< 0.01
Sex (Male vs Female)	0.75	1.49	0.62
Breed*Sex	-3.59	1.79	0.05
***EPA***^***3***^*** (20:5n-3)***			
Breed (Hereford vs Angus)	0.32	0.22	0.16
Breed (Wagyu vs Angus)	0.93	0.20	< 0.01
Sex (Male vs Female)	0.03	0.24	0.92
Breed*Sex	-0.64	0.29	0.03
***DHA***^***4***^*** (22:6n-3)***			
Breed (Hereford vs Angus)	0.19	1.73	0.91
Breed (Wagyu vs Angus)	7.87	1.52	< 0.01
Sex (Male vs Female)	0.86	1.88	0.65
Breed*Sex	-7.12	2.25	< 0.01
***DPA***^***5***^*** (22:5n-3)***			
Breed (Hereford vs Angus)	0.32	1.55	0.84
Breed (Wagyu vs Angus)	6.04	1.36	< 0.01
Sex (Male vs Female)	0.78	1.69	0.65
Breed*Sex	-4.22	2.02	0.04
***EPA* + *DHA***			
Breed (Hereford vs Angus)	0.51	3.21	0.88
Breed (Wagyu vs Angus)	13.91	2.82	< 0.01
Sex (Male vs Female)	1.63	3.49	0.64
Breed*Sex	-11.34	4.18	0.01
***EPA* + *DHA* + *DPA***			
Breed (Hereford vs Angus)	-0.25	6.83	0.97
Breed (Wagyu vs Angus)	27.55	6.00	< 0.01
Sex (Male vs Female)	2.05	7.42	0.78
Breed*Sex	-15.28	8.88	0.09
***ALA* + *EPA* + *DHA* + *DPA***			
Breed (Hereford vs Angus)	-2.89	357.01	0.99
Breed (Wagyu vs Angus)	236.80	313.29	0.45
Sex (Male vs Female)	-367.70	387.60	0.34
Breed*Sex	134.51	462.35	0.77
***tSFA***^***6***^			
Breed (Hereford vs Angus)	-78.43	419.72	0.85
Breed (Wagyu vs Angus)	368.21	368.32	0.32
Sex (Male vs Female)	-465.10	455.68	0.31
Breed*Sex	71.53	543.56	0.90
***tMUFA***^***7***^			
Breed (Hereford vs Angus)	-6.33	28.11	0.82
Breed (Wagyu vs Angus)	64.76	24.67	0.01
Sex (Male vs Female)	5.84	30.52	0.85
Breed*Sex	-47.35	36.41	0.20
***tPUFA***^***8***^			
Breed (Hereford vs Angus)	-7.74	22.37	0.73
Breed (Wagyu vs Angus)	38.28	19.63	0.05
Sex (Male vs Female)	6.54	24.28	0.79
Breed*Sex	-38.85	28.97	0.18
***n-6 PUFA***^***9***^			
Breed (Hereford vs Angus)	2.07	7.71	0.79
Breed (Wagyu vs Angus)	26.71	6.77	< 0.01
Sex (Male vs Female)	-0.55	8.37	0.95
Breed*Sex	-9.81	9.98	0.33
***n-3 PUFA***^***10***^			
Breed (Hereford vs Angus)	-0.32	0.21	0.13
Breed (Wagyu vs Angus)	-0.52	0.18	0.01
Sex (Male vs Female)	0.15	0.23	0.50
Breed*Sex	-0.30	0.27	0.27
***n-6/n-3***^***11***^			
Breed (Hereford vs Angus)	0.06	0.03	0.09
Breed (Wagyu vs Angus)	0.10	0.03	< 0.01
Sex (Male vs Female)	-0.04	0.04	0.34
Breed*Sex	0.05	0.04	0.25

^1^Linoleic acid

^2^α-Linolenic acid

^3^Eicosapentaenoic

^4^Docosahexaenoic

^5^Docosapentaenoic acid

^6^Total saturated fatty acids

^7^Total monounsaturated fatty acids

^8^Total polyunsaturated fatty acids

^9^Omega-6 polyunsaturated fatty acids

^10^Omega-3 polyunsaturated fatty acids

^11^Ratio of n-6/n-3

**Fig. 1:**
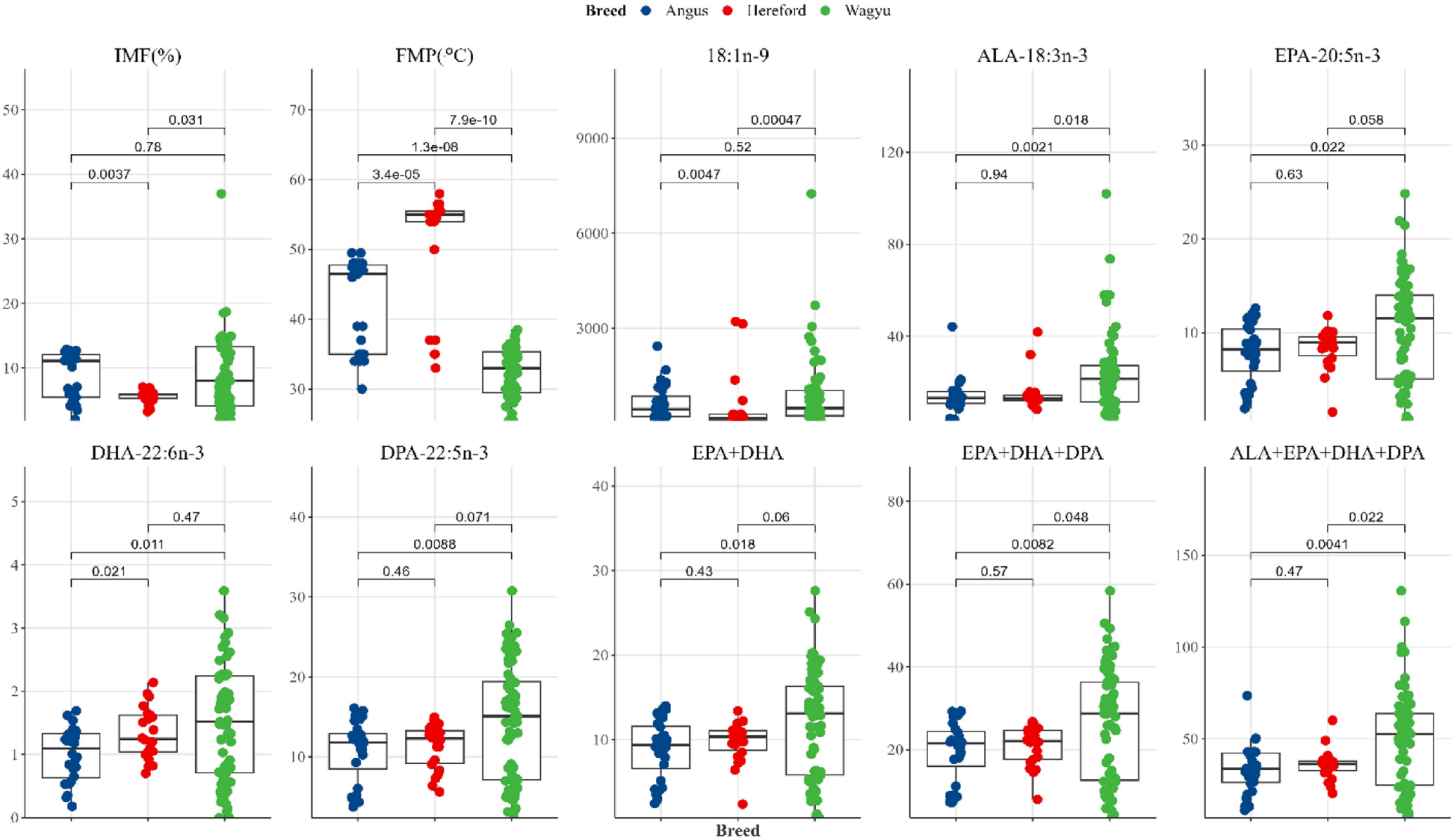
Bonferroni’s adjusted *p*-values for multiple breed comparisons of intramuscular fat (IMF), fat melting point (FMP), oleic acid (18:1n-9), 18:3n-3 (ALA) and long-chain omega-3 polyunsaturated fatty acids (EPA, DHA, DPA) in the *M. longissimus dorsi* muscle of Bowen Genetics Forest Pastoral Angus (  ), Hereford (  ) and Wagyu (  ) cattle. Fatty acids are presented in mg/100g tissue. Significance level set at (*P* < 0.05)

Pairwise multiple breed comparisons with Bonferroni’s adjusted *p*-values in Fig. 1 shows differences between the cattle breeds in IMF, FMP, and LC omega-3 PUFA composition in the *M. longissimus dorsi* muscle. There were differences between Angus and Wagyu in FMP (*P* < 0.05), ALA (*P* < 0.01), EPA (*P* = 0.02), DHA (*P* = 0.01), DPA (*P* = 0.01), and total EPA + DHA + DPA (*P* = 0.01). However, there were no significant differences in oleic acid (*P* = 0.52) and IMF (*P* = 0.78) between Angus and Wagyu. There were no differences in ALA (*P* = 0.94), EPA (*P* = 0.63), DPA (*P* = 0.46), and total EPA + DHA + DPA (*P* = 0.57) compositions between Angus and Hereford. However, differences in IMF (*P* < 0.01), FMP (*P* < 0.05), oleic acid (*P* < 0.01), and DHA (*P* = 0.02) were observed between Angus and Hereford.

**Fig. 2:**
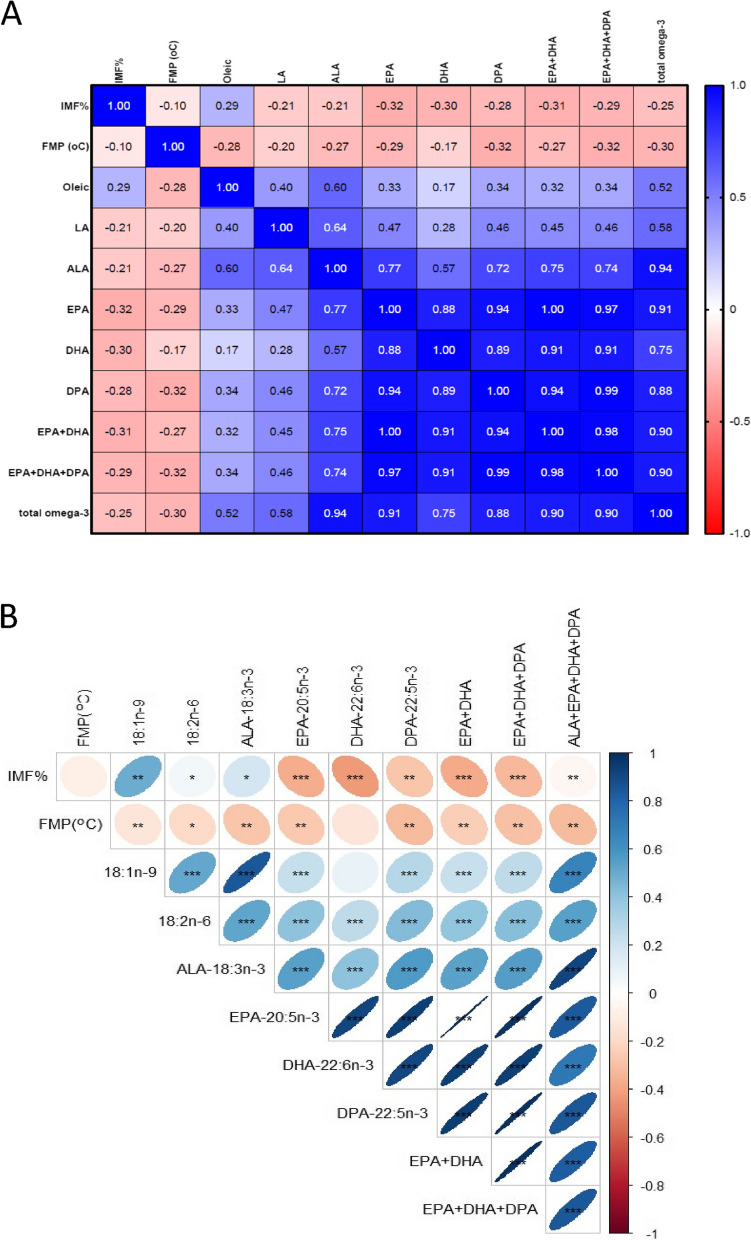
Correlation **A** and *P*-value **B** plots summarizing the relationships between IMF (intramuscular fat), FMP (fat-melting point), and fatty acids of the *M. longissimus dorsi* muscle*.* Blue indicates positive correlations, and red shows negative correlations. Total omega-3 = ALA + EPA + DHA + DPA. Fatty acids are presented in mg/100g tissue. (* *P* < 0.05, ** *P* < 0.01, *** *P* < 0.001)

The correlations between IMF, FMP, oleic acid, LA, ALA, EPA, DHA, DPA, total EPA + DHA, total ALA + EPA + DHA + DPA, and total EPA + DHA + DPA are presented in Fig. 2. Highly positive correlations (*P* < 0.001) between ALA and oleic acid (0.60), EPA (0.77), DHA (0.57), DPA (0.72), EPA + DHA + DPA (0.74), and total omega-3 (0.94), and medium to low correlations between LA and LC omega-3 PUFA EPA (0.47), DHA (0.28), and DPA (0.46) were observed. Similarly, a positive relationship between IMF and oleic acid was evident (*P* < 0.01), while the correlations between IMF and FMP, ALA, EPA, DHA, and DPA were negative (Fig. 2B; *P* < 0.01).

## Discussion

Ensuring that all animals were of yearling age, similar body condition and liveweight, grass-fed, kept under the same management, and muscle biopsy samples taken from the same anatomical site minimized the impact of potential confounding phenotypic factors capable of affecting lipid metabolism attributes. The modern consumers' quest for healthy meat products with adequate health-beneficial LC omega-3 PUFA to minimize the risks and burdens of cardiometabolic and cancer-related mortality [71, 72] has been one of the drivers behind meat producers re-aligning their breeding programs to meet this market demand [1]. Although the easiest and fastest means of modifying the fatty acid composition of ruminant livestock is through dietary supplementation, previous studies had demonstrated that this approach is a temporary and non-cumulative measure, with highly variable and contentious results depending on supplement type and a myriad of other factors [8]. The breed selection criterion on the other hand, presents a cumulative and permanent improvement strategy if breed variation in the trait of interest is sufficiently high. The Angus has become very popular among Australian beef producers since the 1990s due to its intrinsic ability for a relatively faster growth rate than the Wagyu, and its intermediate IMF content compared to the Hereford, Australia's traditional beef breed of choice, renowned for its heavy, high-quality carcasses in both grazing and feedlot production systems [73]. The Wagyu breed has a distinctively high IMF content and is a late maturing breed, making it ideal for the long-fed, high-quality, Japanese beef market [73], specifically bred for high marbling carcasses [74]. The selection of beef cattle with great propensity to produce health-beneficial LC omega-3 PUFA may be the primary strategy by which fatty acid composition can be modified permanently.

### Intramuscular fat and fat melting point

Meat eating quality drives consumer preferences, acceptability, and satisfaction, which all hinge on high IMF and low FMP, as critical drivers of high quality beef consumption. Therefore, beef producers continuously strive to add value to ruminant-derived meat products to meet this rising market demand [74]. In many developed countries, including Australia, IMF is considered as a significant determinant of carcass quality. FMP is associated with the overall palatability of meat [75], because it is an indicator of the degree of saturation in fatty acids, which in turn, impacts meat firmness, processing ease, and consumer appreciation of beef [54, 74]. This study established that the Wagyu breed had the highest IMF (11.1%) and lowest FMP (33.0 °C) in comparison to the Hereford breed with the lowest IMF (5.2%) and highest FMP (55.0 °C). The low FMP of the Wagyu breed in this study agrees with previous reports of Smith et al. [68] and Chung et al. [52] where eight and twelve months old hay-fed Wagyu cattle were reported to have FMP of 35.3 °C and 38.9 °C, respectively. Eight and twelve months old grass-fed Angus cattle had FMP of 37.9 °C and 42.8°C, respectively [52]. Australian Angus x Hereford crossbreds were reported to have FMP of 47.2°C [76], compared to purebred Japanese black steers at 14 months of age with a melting point of 35.5 °C, which decreased to 21.2 °C when they were 28 months old [74].

The main fatty acids found in beef are stearic acid (18:0), palmitic acid (16:0) and oleic acid (18:1), which are known to affect the hardness, softness and FMP of fat [20, 77–79]. Soft fat in beef has a low melting point because of its high unsaturated fatty acids content, mainly MUFA and PUFA, whereas hard fat has a high melting point because of its high SFA content [68, 78]. The double bonds in MUFA and PUFA can be more easily broken, resulting in comparatively lower FMP than the more stable SFA single bonds that are more difficult to break, hence resulting in high FMP [61].

The melting points of stearic and palmitic acids are 69.6 °C and 62.9 °C, respectively, hence they are associated with higher FMP [80]. In contrast, oleic acid melts at 13.4 °C, hence its high concentration in beef fat leads to a low FMP [78]. The breed differences in FMP observed in this study may also be due to the stearoyl-CoA desaturase (SCD) enzyme activity and gene expression. The SCD gene encodes the rate-limiting enzyme that catalyzes the synthesis of MUFA from SFA [78]*.* Chung et al. [81] reported 20% greater SCD activity and 70% more SCD mRNA in Wagyu than in Angus adipose tissues. MUFA constitute soft fat which has a low melting point because it consists of high unsaturated fatty acids content with double bonds that are easy to break under low heat.

Previous studies in Australian and Japanese beef cattle showed significant increases in FMP as the concentration of stearic acid increased in the adipose tissue. Japanese Black cattle raised under Japanese conditions contained only 8% stearic acid of total fatty acids, with an average melting point of 22.8 °C [75]. However, under Australian feedlot management conditions, stearic acid increased to 25% with an average FMP of 45.1 °C [68]. Chung et al. [52] and Wood et al. [79] reported a strong relationship between FMP and stearic acid in addition to demonstrating that different production systems diversely contribute to beef cattle fat deposition, fatty acid composition, and associations with FMP.

A previous report on sensory evaluation found that the IMF content of *M. longissimus dorsi* of Japanese Black steers ranged from 23.2%—48.6% and significantly increased juiciness, fattiness, and tenderness [82, 83]. In the current study, the IMF was less than the 25%—40% range previously reported in grain-fed, purebred Wagyu in Japan [82, 84, 85], possibly due to dietary and age differences, as the Australian pasture-based Wagyu in the current study, were yearlings fed solely on pasture, hence, the observed lower IMF is expected [73]. It has been reported that grain-fed beef cattle increase their IMF content faster than their pasture-fed counterparts [86]. Therefore, most Wagyu beef cattle aimed at the Japanese market are intensively fed on high energy-dense grains in the feedlot to produce intramuscular lipid concentrations of over 30% [87, 88]. This is because consumers prefer marbling with health-beneficial LC omega-3 PUFA and adequate crude protein [89]. The present results are similar to a previous study conducted in an Australian pasture-based production system where the IMF in Wagyu ranged from 7.8—17.5%, and 5.2—9.9% in Angus [64]. Hereford-sired steers born to Angus, Holstein–Friesian, and Jersey cows had IMF percentages ranging from 2.7% to 5.8% in the New Zealand pasture production system [90]. In Argentina, pasture-fed Angus and Hereford had IMF percentages of 3.1% and 2.4%, respectively [40]. Our results corroborate previous studies that reported pasture-fed beef cattle with lower marbling compared to grain-fed cattle, regardless of breed [86]. The IMF of Angus, Hereford, and Wagyu beef cattle breeds in the current study were all above the 3% threshold required in Australia for classifying beef as high meat eating quality [91, 92].

### Linoleic, α-linolenic, oleic acids and their correlations

The current fatty acid composition results demonstrate the dominance of oleic acid in the *M. longissimus dorsi* muscle of Wagyu and Angus followed by Hereford in that order (Fig. 1 and Table 1). This agrees with other studies that reported similar dominance of oleic acid in the muscles of pasture-fed Wagyu [93], Angus [94], and Hereford x Angus [95]. Positive relationships between fatness and oleic acid in ruminants have also been reported [96, 97]. In the present study, both Angus and Wagyu had greater IMF and oleic acid contents than the Hereford. A direct link exists between low FMP and oleic acid with impacts on the overall palatability and acceptability of red meat by consumers [52, 74]. A low FMP allows the fat to melt in the mouth during consumption and contributes to the unique taste of beef [74, 98]. The type of production system also affects the concentration of oleic acid in the muscle of beef cattle [64]. High and low concentrations of oleic acid in feedlot and grass-fed systems, respectively, were reported by Smith et al. [75].

The concentration of oleic acid in the Wagyu differs from other beef cattle breeds because of its high genetic disposition to increased SCD enzyme activity and gene expression in the adipose and muscle tissues [75], which has been reported to have a very high heritability in Japanese Black cattle [99]. In the current study, the concentration of oleic acid in pasture-based yearling Angus and Wagyu were comparable. This could be explained by the fact that we analysed muscle biopsy samples where the activity of SCD is reported to be low compared to the adipose tissue [100–103]. Also, it has been previously reported that pasture feeding depresses the activity of SCD and reduces marbling score [81, 104, 105]. Furthermore, although there is a rapid increase in subcutaneous adipocyte volume between birth and weaning, de novo fatty acid biosynthesis is comparatively gradual, slower, and less associated with any significant change in ∆9 desaturase gene expression at the growing stages of yearling calves [75]. Sturdivant et al. [106] reported on the role of SCD in the conversions of stearic, palmitic, and myristic acids to oleic acid in muscle and adipose tissues that support the claim of a genetic basis for oleic acid biosynthesis in ruminants [106]. In this present study, no significant differences were observed in the concentrations of LA in grass-fed Angus, Hereford and Wagyu. However, the Wagyu had nearly twice as much ALA as Angus and Hereford, thus suggesting a possible genetic basis for this variation [59]. Other studies have reported lower LA and ALA values in Angus and Hereford than in the present study [40, 64].

### Eicosapentaenoic, docosahexaenoic and docosapentaenoic acids

The highest DPA and EPA + DHA + DPA LC omega-3 PUFA contents were in Wagyu, except for EPA and DHA, where no clear differences were observed between Wagyu, Angus and Hereford. The levels of EPA, DHA, and DPA observed in the current study are similar to those reported by Frank et al. [64] and Bermingham et al. [93]. Other reports have shown that LC omega-3 PUFA concentrations are higher in pasture-finished than feedlot-finished production systems [107–109], primarily due to the high concentration of ALA in pastures [19, 110]. This suggests that the pasture-based production system could provide a cheap and long-term solution for producing beef with consistent meat eating quality and improved health-promoting LC omega-3 PUFA. This result will sit very well with health-conscious consumers who are willing to pay a premium for healthy beef [1, 16]. At 28.8 mg/100g, the Wagyu was closest to the Australia and New Zealand recommended “source level” threshold of 30 mg/100g tissue of health-beneficial LC omega-3 fatty acids content [69]. Although seafood is the richest dietary source of LC omega-3 PUFA [111], over-exploitation of fish stocks, individual preference, and cultural inculcation limit the use of seafood as a sustainable source of LC omega-3 PUFA [23–25]. In Australia, the recommended total DHA + EPA + DPA levels are 90 and 160 mg/day for women and men, respectively [69]. Since beef and veal consumption is estimated at 49.5 g/day [33], the cattle breeds evaluated in this study can provide approximately 12–16% of the recommended DHA, EPA and DPA intake for women and 7–9% for men. Therefore, red meat will continue to be an important source of LC omega-3 PUFA [28] that may help meet the dietary deficit caused by low consumption of seafood. Although an increase in muscle PUFA composition can potentially reduce meat oxidative stability [112], the storage of vacuum-packaged meat at 1.5 ± 0.8 °C has been reported to maintain the oxidative stability of striploin muscle samples obtained from Australian grass-fed cattle for up to 20 weeks [113], hence, consumers can access beef with high composition of the health claimable PUFA at a reduced risk of lipid oxidation.

The Wagyu breed has a fatty acid profile that is different from other cattle breeds, because of its comparatively higher genetic predisposition to produce more MUFA than other bovine breeds [49, 53, 63, 74, 98, 114–116]. The heritability of MUFA and PUFA in Wagyu cattle has also been reported to be very high at 0.68 and 0.47, respectively [99]. Breed affects the concentration of unsaturated fatty acid in beef cattle by influencing the activities and expressions of lipogenic genes such as the SCD, fatty acid binding protein (FABP) and fatty acid synthase (FAS) [8, 52, 86]. Therefore, the summation differences in total EPA + DHA + DPA seen in Table 1 could be as a result of variation in genetic predisposition between the breeds, because we ensured that all potential confounding phenotypic factors capable of affecting lipid metabolism were minimized in the present study. The pasture feeding of beef cattle also influences the concentrations of linoleic and α-linolenic acids [8]. Previous studies have shown that the duodenal flow of α-linolenic acid (a precursor for the de novo synthesis long-chain polyunsaturated fatty acids) in pasture-fed cattle increases, while that of oleic acid simultaneously decreases due to a decrease in the activity of SCD [117, 118]. Therefore, the genetic selection of beef cattle breeds in a pasture-based system with greater genetic propensities to synthesize LC omega-3 PUFA may be the solution for a permanent, sustainable, long-term, and cumulative approach to modifying fatty acid composition in ruminants.

### Gender and interactions with breed

The composition of fatty acids and IMF in the skeletal muscle of cattle is markedly affected by gender [119]. The main effect of gender in the present study was observed for IMF, FMP, the compositions of ALA, DHA, DPA, EPA + DHA, EPA + DHA + DPA and ALA + EPA + DHA + DPA, where yearling heifers performed better than yearling bulls in all categories. These data are consistent with the previous results showing that heifers have significantly higher fat content and the composition of fatty acids compared to intact bulls [120]. A study conducted by Carrilho et al., [121] found that castrated bulls produced more IMF and the composition of oleic acid and DPA than intact bulls. Judge et al., [122] found that meat quality of intact bulls was inferior to that of both castrated bulls and heifers with comparable results between the latter two genders, suggesting that the effect could be a result of differences in sex hormones [123] between the intact bulls and heifers. A study by Zhang et al., [124] found that testosterone levels were significantly reduced in castrated bulls, whereas the IMF content and triglycerides (TGs) were significantly increased compared to intact bulls. The effect of hormones, testosterone and oestrogen, in muscle tissues has been well documented [125]. Oestrogen plays a major role in the regulation of energy metabolism pathway, including glycolysis, fatty acid β-oxidation synthesis and glucose transportation [126]. Testosterone on the other hand, downregulates the activities of glycerol-3-phosphate dehydrogenase and adiponectin secretion, an adipose-specific secretory protein, in the differentiation of bovine intramuscular adipocytes [127, 128]. A previous study found that castrated Korean cattle expressed the lipogenic genes *acetyl-CoA carboxylase and fatty acid synthase*, but suppressed the expressions of the lipolytic genes *adipose triglyceride lipase and monoglyceride lipase* in the *M. longissimus dorsi* [129]. The results in the present study suggest that gender contributes to an improved meat quality through the regulation of hormones and lipogenic genes to increase lipid uptake and lipogenesis, and should be a considered strategy for improving the composition of LC omega-3 PUFA, IMF and FMP in Angus, Hereford and Wagyu in a pasture-based production system. The significant interaction between breed and sex observed in FMP, ALA, EPA, DHA, DPA and EPA + DHA (Table 3) suggest that hormonal differences between genders would vary between breeds and this interaction should be taken into consideration when selecting for FMP, IMF and health-beneficial LC omega-3 PUFA in the *M. longissimus dorsi* muscle of grazing Angus, Hereford and Wagyu beef cattle.

## Conclusion

Taken together, the outcomes of the current study conclusively demonstrate that in a pasture-based production system, breed, sex and their interactions were significant sources of variation in IMF, FMP and LC omega-3 PUFA content in yearling bulls and heifers of the same age and routine management. Yearling heifers produced more IMF with lower FMP, and higher LC omega-3 PUFA content than yearling bulls. At 28.8 mg/100g tissue, the Wagyu breed produced *M. longissimus dorsi* muscle with the highest LC omega-3 PUFA content, and was the closest to attaining the Australia and New Zealand recommended “source level” threshold of 30 mg/100g tissue. These findings also suggest that the Bowen Genetics Forest Pastoral beef cattle studs are well positioned in the international research context to improve and provide important alternative sources of LC omega-3 PUFA that can be used to cover the deficit in the health claimable fatty acids in Western diets.

## Methods

The reporting in the manuscript follows the recommendations of Kilkenny et al. [130] in the ARRIVE guidelines (2.0 Essential 10 list for animal research). The use of animals and protocols performed in this study were approved by the James Cook University Animal Ethics Committee and were conducted in accordance with the Animal Care and Protection Act for the Australian Code for the Care and Use of Animals for Scientific Purposes (Ethics Approval Number A2724). The owners of the farm consented to the use of their herds as experimental animals for this study.

### Animals and management

In this on-farm study, 30 Angus (14 males, 16 females), 22 Hereford (19 males, 3 females), and 75 Wagyu (25 males, 50 females) beef cattle, progeny of 15 sires in total, comprising 5 sires from each breed, raised, and maintained as self-replacing purebred herds at Bowen Genetics Forest Pastoral stud, Barraba, New South Wales, Australia, were utilized. As opposed to an on-station experimental design where equal numbers of animals are possible, this was an on-farm experiment with variable number of available cattle, hence an unequal distribution of sample sizes between breeds. The animals grazed with their dams until they were weaned at 6–8 months and pasture-fed on ryegrass, kept in the same property but separated by breed and sex into six herds. The six paddocks were monitored regularly to ensure sufficient ryegrass pasture allowance. At sampling, the cattle were 12 ± 2.43 months old, of similar body condition scores (3.0–3.5) and 450–455 kg liveweight.

### Muscle biopsy sampling, fatty acid, intramuscular fat, and fat-melting point analyses

The *M. longissimus dorsi* biopsy samples were obtained from the interface of the 12th and 13th ribs, and the sampling procedure was similar to that described previously by Malau-Aduli et al. [51], Pewan et al. [131], and Otto et al. [132], while the laboratory procedures for the determination of fatty acid profile, IMF, and FMP have been extensively described in our previous publications [22, 50, 57]. Briefly, the hair from around the 12th and 13th ribs of the experimental animals kept in a crush was clipped. A disinfectant, alcohol/chlorohexidine solution was applied at the surgical site. About 20 mL of a local anaesthetic, lignocaine, was injected at the surgical site before 2-5g of muscle biopsy was collected with a sterilized scalpel. The incision site was stitched, and antibiotics administered to prevent infection. One gram of the muscle biopsy sample was homogenised, transferred to a labelled 50 mL plastic tube containing 20 mL of CHCl_3_:MeOH (2:1) solvent and 5 mL of 10% KCl to analyse for IMF content. The extracted IMF was placed in an oven at 100 °C for about 1–2 min to melt. The melted IMF was sucked into a thin capillary tube using air suction and gradually heated in a test tube containing cold water and a bulb thermometer to estimate the FMP. A single-phase overnight extraction technique utilizing CHCl_3_:MeOH:H_2_O (1:2:0.8 v/v) was used to extract total lipids from the remaining 1 g of un-homogenized muscle tissue samples, followed by phase separation with the addition of CHCl_3_:Saline-Milli-Q H_2_O (1:1 v/v). The lipids were separated into classes by thin-layer chromatography (TLC). An aliquot of the lipid extract was utilized for transmethylation with MeOH:CHCl_3_:HCl (10:1:1 v/v). Fatty acid methyl esters (FAME) were extracted thrice using hexane (4:1 v/v). An internal standard (C19:0) of a known concentration was added in a 1500 μL vial encompassing the extracted FAME. The fatty acids content was calculated as follows: FA mg/100 g = (Total lipid) × (LCF [0.912]) × ([%FA]/100) × 1000, where 0.912 was the lipid conversion factor (LCF).

### Statistical analysis

The R statistical software version 4.0.2 (R Foundation for Statistical Computing, Vienna, Austria) was used to perform data analysis. Summary statistics were initially computed to scrutinize the data for entry errors and normality testing. The data were found to be non-normally distributed, hence, a non-parametric approach was applied. Descriptive summary statistics of fatty acid composition, IMF, and FMP were presented as medians and inter-quartile ranges. Breed, sex and sire within breed differences in fatty acid composition were statistically tested using the Kruskal–Wallis test and Wilcoxon tests (Mann–Whitney) with Bonferroni’s adjusted *P*-value across groups. The relationships between IMF, FMP, and fatty acids were evaluated using the Spearman correlation analysis. Statistical inference was set at a *P* < 0.05 level of significance. Furthermore, the effect of breed, sex and their interactions on fatty acid composition were tested using linear regression models.

## Data Availability

The datasets generated and/or analysed during the current study are not publicly available due to contractual confidentiality clause obligation, but are available from the corresponding author on reasonable request.

## Abbreviations

LC omega-3PUFA: Long-chain omega-3 polyunsaturated fatty acids
FMP: Fat-melting point
IMF: Intramuscular fat
LA: Linoleic acid
ALA: α-Linolenic acid
EPA: Eicosapentaenoic
DHA: Docosahexaenoic
DPA: Docosapentaenoic
tSFA: Total saturated fatty acids
tMUFA: Total monounsaturated fatty acids
tPUFA: Total polyunsaturated fatty acids
n-6: Omega-6 fatty acids
n-3: Omega-3 fatty acids
ARRIVE: Animal Research: Reporting of In Vivo Experiments
CHCl3: Chloroform
MeOH: Methanol
TLC: Thin-layer chromatography
FAME: Fatty acid methyl esters
LCF: Lipid conversion factor
KCL: Potassium chloride
H_2_O: Water
FA: Fatty acid
